# Vegetation extraction through UAV RGB imagery and efficient feature selection

**DOI:** 10.1371/journal.pone.0322180

**Published:** 2025-05-09

**Authors:** Junliang Dong, Jian Zhang, Suo Zhang, Zhiyong Yu, Ziheng Song, Tianya Meng

**Affiliations:** 1 Shenhua Group Xinjie Energy Co. Ltd, Ordos, China; 2 State Key Laboratory of Water Resources Protection and Utilization in Coal Mining, Beijing, China; 3 School of Mining and Geomatics Engineering, Hebei University of Engineering, Handan, China; 4 Satellite Application Center for Ecology and Environment, Ministry of Ecology and Environment of People’s Republic of China, Beijing, China; Universidade do Vale do Rio dos Sinos, BRAZIL

## Abstract

Accurate identification of vegetation in mining areas is crucial for conducting pre-mining ecological assessments and post-mining ecological monitoring. However, the vegetation in the mining area is always highly heterogeneous including both field crops and naturally scattered growing vegetation, which brings great challenges for fine vegetation mapping. Feature combinations are an important factor to influence the vegetation mapping. Thus, to effectively identify the vegetation, this study utilized an unmanned aerial vehicle (UAV) RGB image to extract vegetation indexes and textures, and then selected features based on standard deviation and difference coefficient. By integrating selected optimal features with RGB images, different combinations were constructed and classified using Support Vector Machine (SVM). The results demonstrated that the combination of RGB and all selected features yielded the highest accuracy, followed by the combination of RGB and a single type of texture, and then the combination of RGB and VIs, which indicated that texture features were more important than VIs for vegetation identification. The OA and Kappa for the best combination were 87.76% and 0.8351 for study area A, and 88.74% and 0.8505 for study area B, indicating the effectiveness of the adopted method. Besides, compared with the commonly used random forest (RF) feature selection method, the adopted method avoided complex parameter settings and constructed a superior optimal combination, which further proved the simplicity and effectiveness of difference coefficient-based feature selection methods for vegetation classification in highly heterogeneous environments, contributing to more accurate ecological assessments and monitoring.

## Introduction

The coal mining region in Ordos, Inner Mongolia, serves as a crucial source of energy for the northwest region of China. The Xinjie Taigemiao mining area, situated in Ordos, falls under the jurisdiction of Ejin Horo Banner and Wushen Banner. This area features exceptional overall construction conditions, including an ideal location and ample coal reserves. It is a large-scale, undeveloped integrated coalfield that can serve as a model for the creation of a large-scale, intensive, and intelligent mining area. To effectively analyze the characteristics of regional ecological changes, post-mining land reclamation, and ecological restoration, it is necessary to accurately determine land use and surface vegetation distribution.

With the development of remote sensing technology, it has gradually become an effective method for vegetation monitoring and land use investigation due to the advantage of large coverage area and strong detection capabilities [1–3]. However, when the study focused on the working face of mining areas, it required the high-resolution images with expensive cost and highly dependent on weather conditions during satellite transit [4], which made it difficult to access the appropriate data for vegetation identification. Recently, UAV remote sensing has been widely used since it is with high flexibility, high spatial resolution and low susceptibility to weather and cloud cover [5,6]. Scholars have applied the UAV-based RGB image to distinguish different ground objects such as tree mapping [7,8] and crop identification [9, 10], and proved their feasibility and effectiveness. Compared with other sensors, they are more affordable and therefore increasingly become the choice for vegetation study.

Currently, there are mainly two types of vegetation mapping methods based on UAV RGB imagery. The first one take use of vegetation indexes (VIs) to increase the differences between the vegetation and non-vegetation, and then extracted the vegetation by threshold segmentation. For example, Wang et al. [11] constructed the Visible-band Difference Vegetation Index (VDVI) to achieve high precision recognition of healthy green vegetation. Zhang et al. [12] designed a new Green-Red Vegetation Index (NGRVI) according to the characteristics of arid and semi-arid vegetation and realized the effective extraction. Although this type of methods is simple to operate, they are often applied to single type of land cover identification.

The second type of method is based on image enhancement and texture feature extraction. Li et al. [13] and Zhou et al. [14] proposed object-based image analysis (OBIA) method to identify shrub species and wetland vegetation respectively, they first segmented the image into homogeneous regions, and then used feature extraction or selection methods to integrate feature combinations of spectrum, VIs and textures to differentiate objects. Although OBIA are always adopted for the high-resolution image, they face the challenges in the process and determination of optimal scale [15], and the accuracy is closely related to the experience and prior knowledge of image operators [16]. Thus, considering the simplicity and effectiveness, pixel-based analysis methods are still one of the most used methods [16,17]. The feature combinations are an important factor to influence the vegetation mapping results, and random forest (RF) have been used for feature selection [17,18]. However, it needs to determine the optimal number of trees, predictor variables and features by trial and error. Feature selections based on coefficient of variation are simple and efficient methods which showed better performance compared with some state-of-the-art methods [19,20]. Han et al. [21] analyzed the variation coefficients and difference coefficients of RGB texture between maize and other vegetation and then selected the mean of green band and the homogeneity of blue band to identify maize. Guo et al. [22] used above methods to analyze HSV(Hue, Saturation and Value) and 24 texture features, and finally selected brightens, saturation and red second order moment to identify farmland crop such as grape, maize and cotton. Hu et al. [23] also applied the same method to extract the subsided cultivated land. Although above studies showed good performance for identification, they only focused on the farmland crop with homogeneous spatial distribution, leaving a gap for high heterogeneity environments such as mining areas with field crops and naturally growing vegetation. Besides, VIs are effective features for vegetation identification [24–26], but they were not considered in the feature selections.

To address the above issues, this study explored the effectiveness of vegetation mapping in highly heterogeneous areas with feature selection method based on standard deviation and difference coefficients, which could avoid complex parameter settings of commonly used feature selection methods. This study integrated UAV-based RGB imagery with selected vegetation indices (VIs), RGB textures, and HSV textures, and then used support machine vector (SVM) to classify on the different constructed feature combinations to identify the vegetation of Xinjie Taigemiao mining area. The contributions of the study are as follows: (1) to determine whether the difference coefficient based feature selection method can apply to the extraction of non-crop plants with high heterogeneity; (2) to validate the effectiveness of incorporating VIs into the feature; and (3) to explore the optimal combinations of RGB image with other features. This work could provide new insights for ecological analysis of before and after mining such as diversity investigation and mining impact evaluation.

## Materials and methods

### Study area and data acquisition

**Fig 1:**
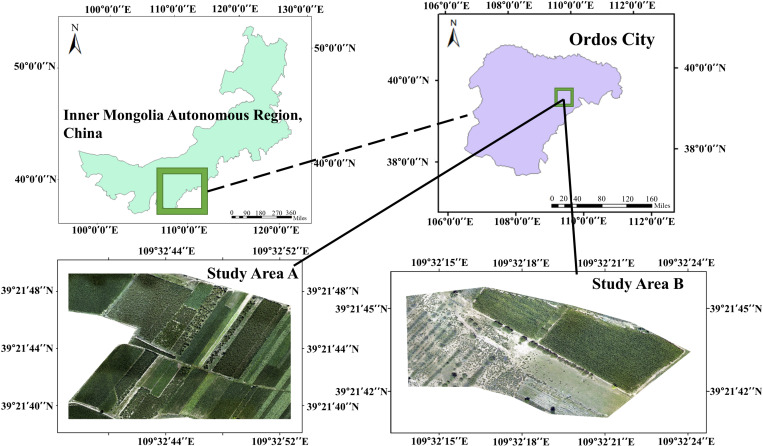
Visible light images of the study area. A presents the data of experimental region, B presents data of verified region. Map was created using ArcGIS from Esri (http://www.arcgis.com). The base map images were author-owned RGB image data acquired from UAV surveys.

**Fig 2:**
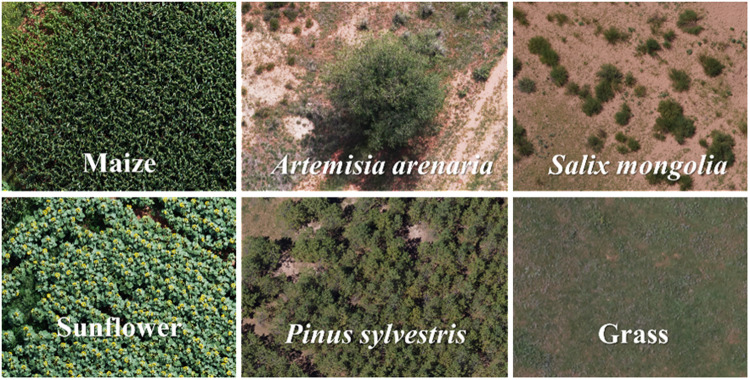
The main vegetation in the study area.

This paper selected the initial working face of Xinjie Taigemiao mining area as the study area as shown in Fig 1. The vector of the administrative map was from Resource and Environmental Science Data Platform: https://www.resdc.cn/DOI/DOI.aspx?DOIID=121. This region is located in the ecological functional area of typical grassland desertification control of Ordos Plateau, and the landscapes are mainly the hills and undulating plains. It has inland semi-arid climate with heterogeneous vegetation such as crop, trees and shrubs. This study was carried out in two experimental areas. The study area A covers an area of about 9.9 × 104 m2, and the main land cover types were *Zea mays* (maize), *Helianthus annuus* (sunflowers), *Pinus sylvestris*, grasslands and bare land (Fig 2). Among them, the *Pinus sylvestris* varied greatly in density and crown size. The study area B covers an area of about 2.4 × 104 m2, and the main land covers area B were maize, *Artemisia arenaria*, *Salix mongolia*, grasslands and bare land in which *Artemisia arenaria* and *Salix mongolia* were scattered distribution.

The DJI M600 were selected as the UAV platform which was equipped with the Phase One IXM 100 MP visible light digital camera to acquire the high resolution RGB image with a spectral range of 0.4–0.7 µm. The adopted sensor type of the camera was CMOS with dynamic range of 83dB. The camera was with a range of specifically designed RSM lenses from 35 mm to 300 mm, and max field of view was 63°. The UAV flight height was set to 500 meters, and the overlaps were 80% and 70% in the flight direction and in the lateral direction, respectively. The photo interval was 3 seconds. The data was acquired in September 2022 at which time most of sunflowers were blooming. The orthoimages with 10.0 cm/pixel spatial resolution were generated with the software of Agisoft Metashape 1.8.4. and stored in the TIFF format. Each band of RGB image contained 8-bit information with a value range of 0–255.

### Workflow overview

**Fig 3:**
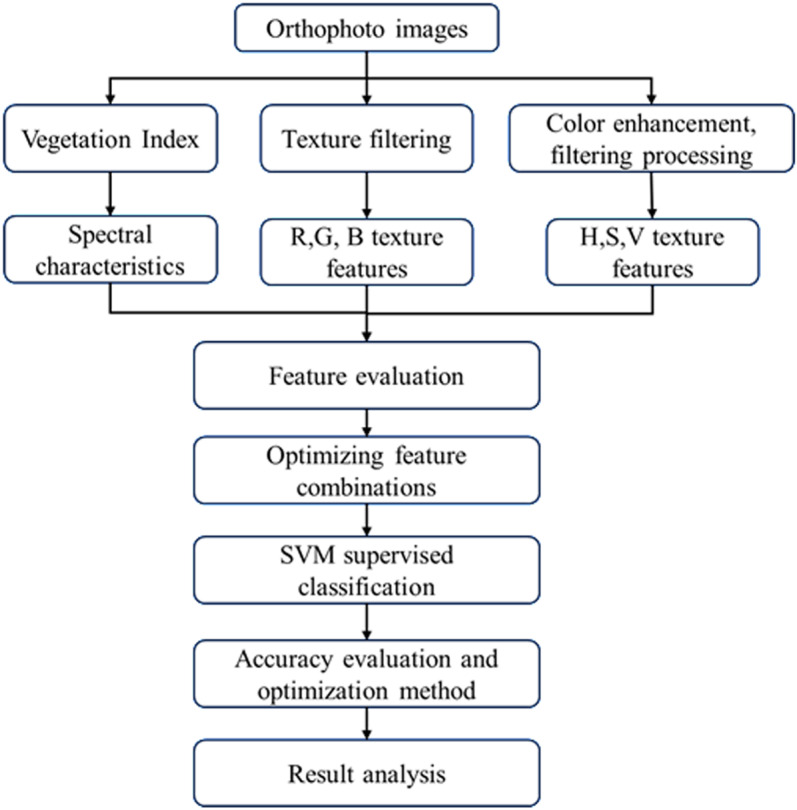
Workflow of this study.

Since UAV RGB image only contains the red, green, and blue band, it is difficult to distinguish different vegetation with the RGB color information. Therefore, this paper extracted VIs, RGB textures and HSV textures to improve the differentiation between the vegetation, and the extracted features were normalized to make them comparable. Then, the standard deviation and inter-class difference coefficient between different vegetation were compared and analyzed to select optimal features and construct the feature combinations with RGB image. This paper constructed the following combinations: RGB & optimal VIs, RGB & optimal RGB texture features, RGB & optimal HSV texture features, and RGB & optimal VIs & optimal RGB texture features & optimal HSV texture features. Then SVM was conducted based on the combinations to identify the vegetation. Visual interpretation combining with confusion matrix was used to evaluate the accuracy to determine the optimal feature combinations. The workflow of the study was shown in Fig 3.

### Feature extraction

#### Vegetation index extraction.

Vegetation indexes have been proved effective for vegetation identification. However, compared with multispectral images, UAV visible light images could not apply traditional indexes such as Normalized Difference Vegetation Index (NDVI) since they did not contain near-infrared band. In this situation, scholars have constructed varieties of indexes focusing on the RGB images to increase the differentiation between the vegetation and non-vegetation. To explore the feasibility of UAV visible light images for vegetation classification and identification, commonly used visible light vegetation indexes were used including Red Green Ratio Index (RGRI) [27], Normalized Green Blue Difference Index (NGBDI) [28], Normalized Green Red Difference Index (NGRDI) [29], Excess Green Index (EXG) [30], and Vegetation Difference Index (VDVI) [11], as shown in Table 1.

**Table 1:** Expression for the visible light vegetation index.

Index	Abbreviation	Formula
Red Green Ratio Index	RGRI	RGRI*=*$\frac{\rho_{red}}{\rho_{green}}$
Normalized Green Blue Difference Index	NGBDI	NGBDI*=*$\frac{\rho_{green}-\rho_{blue}}{\rho_{green}+\rho_{blue}}$
Normalized Green Red Difference Index	NGRDI	NGRDI*=*$\frac{\rho_{green}-\rho_{red}}{\rho_{green}+\rho_{red}}$
Excess Green Index	EXG	EXG*=*$2\times \rho_{green}-\rho_{red}-\rho_{blue}$
Vegetation Difference Index	VDVI	VDVI*=*$\frac{2\times \rho_{green}-\rho_{red}-\rho_{blue}}{2\times \rho_{green}+\rho_{red}+\rho_{blue}}$

#### Texture extraction based on RGB image.

Textures can reveal the detailed structures of target objects, and are one of the most important features in the remote sensing images. They could reflect the homogeneity and were used to increase the phenomena discrimination of “same object different spectrum” and “same spectrum foreign body” combining with spectral features, thereby improving vegetation classification accuracy [23,24]. To extract the textures of RGB image, the ENVI software was used to perform co-occurrence measures and default parameters were adopted as follows. The filter window size was set to 3 × 3, and the changes in the spatial correlation matrices X and Y were set to 1. The gray level was set to 64. The extracted texture features included the mean, variance, homogeneity, contrast, dissimilarity, entropy, second moment, and correlation of the R, G, and B bands, resulting in a total of 24 features.

#### Texture extraction based on HSV image.

**Fig 4:**
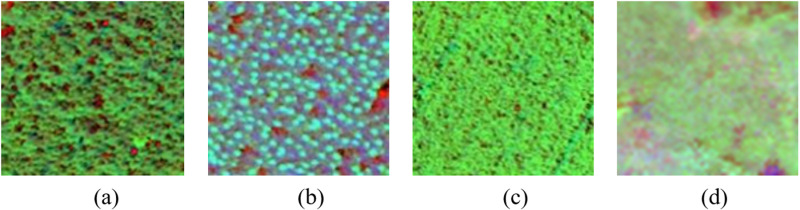
Sample of different vegetation in HSV. (a) Pinus sylvestris, (b) Sunflower, (c) Maize, (d) Grass.

HSV color space consists of hue, saturation, and brightness values. The three components of RGB image always highly correlate with each other, and the conversion from RGB space to HSV space could decouple the chromaticity (H and S) and the brightness (V) [31], which could enhance the readability of an image and was proved useful for object identification and extraction. Thus, the color space transform was performed as shown in Fig 4. It could be observed that there were obvious differences in hue, saturation, and texture between different vegetation types. Therefore, texture filtering was applied to the converted image and the set was the same as the extraction of RGB texture. Then total of 24 texture features were obtained for the H, S, and V components.

### Feature selection

Feature analysis and selection were conducted using the mean, standard deviation (*S*), and inter-class difference coefficient $D_{W}$) of different vegetation samples in the images. The calculation formulas were as follows [32]:


$$S=\sqrt[{\text{2}}]{\sum_{i=1}^{n}{{\text{(x}}_{\text{i}}\text{-x)}}^{\text{2}}\text{/n}}$$ (1)

$$D_{w}\text{=}\frac{X_{1}\text{-}X_{2}}{X_{2}}\times 100\%$$ (2)

where $x_{i}$ represents the sample grayscale value of same class, *n* is the number of samples, x is the sample mean, $D_{w}$ is the inter-class difference coefficient, $X_{1}$ is the sample mean value of one class, and $X_{2}$ is the sample mean value of another class.

The standard deviation reflects the degree of dispersion of feature. The lower the degree of dispersion, the more conducive to use this feature to extract the object. The inter-class difference coefficient reflects the differences between classes. The higher the difference coefficient, the easier to distinguish the different vegetation.

This study only compared and analyzed the standard deviation and difference coefficient among vegetation since the obvious spectral differences between bare land and vegetation made it easy to distinguish. The specific selection method was as follows: Firstly, the difference coefficients between first class and other classes were sorted in reverse order, and features with difference coefficients greater than 70% were selected. Then, the standard deviations of the first class were sorted, and features with standard deviations less than 30% were chosen. This process was repeated until all the classed were compared and analyzed, then the feature combinations for distinguishing different vegetation were determined.

### Vegetation identification

SVM classification is a commonly used machine learning method which can automatically find the support vectors with significant discriminative power for classification. SVM constructs the optimal hyperplanes to maximize class intervals through kernel functions which maps linearly inseparable data to high-dimensional space. It has the characteristics of simple structure, strong adaptability and global optimality, and can effectively solve problems such as high-dimensional features, nonlinearity, over learning and uncertainty. Previous study showed that SVM could achieve good accuracy in vegetation extraction [33], and the radial basis kernel function was more suitable for distinguishing different types of crops [34]. Therefore, this paper used the radial basis kernel function of SVM to distinguish the vegetation based on different optimal feature combinations.

## Results and analysis

### Feature selection result

#### Selection of VIs.

Table 2 showed the standard deviation and difference coefficient of the VIs for different vegetation of study area A. It could be found that the difference coefficients vary greatly, for example, the maximum difference coefficient between sunflower and maize is 59.31%, and the minimum value is 2.47% which indicated that not all features were conducive to the identification and analysis of vegetation. According to the feature selection method, this paper first determined the features to distinguish maize and *Pinus sylvestris*. It could be found that RGRI have largest difference coefficient and a smaller standard deviation which indicated that RGRI could be used to distinguish between maize and *Pinus sylvestris*. Similarly, it was found that RGRI could distinguish between maize and grassland, and EXG could distinguish between maize and sunflower, between *Pinus sylvestris* and sunflower, and between *Pinus sylvestris* and grassland. Therefore, RGRI and EXG were selected as optimal VIs for the study area A. This paper performed the same actions to the study area B and the result showed that optimal VIs was RGRI and NGRDI.

**Table 2:** Standard deviation and difference coefficient of VIs between different vegetation %.

Indexes	Maize				*Pinus sylvestris*				Sunflower				Grass			
*S*	$D_{W}$ *Pinus sylvestris*	$D_{W}$ Sunflower	$D_{W}$ Grass	*S*	$D_{W}$ Maize	$D_{W}$ Sunflower	$D_{W}$ Grass	*S*	$D_{W}$ Maize	$D_{W}$ *Pinus sylvestris*	$D_{W}$ Grass	*S*	$D_{W}$ Maize	$D_{W}$ *Pinus sylvestris*	$D_{W}$ Sunflower
VDVI	0.15	13.00	−2.41	7.45	0.15	−11.51	−13.64	−4.92	0.13	2.47	15.80	10.11	0.18	−6.93	5.17	−9.18
RGRI	0.12	−32.99	−14.91	−38.01	0.14	49.23	26.98	−7.50	0.15	17.52	−21.25	−27.15	0.22	61.32	8.10	37.27
NGRDI	0.13	16.01	4.78	21.01	0.15	−13.80	−9.68	4.31	0.16	−4.56	10.71	15.49	0.23	−17.36	−4.13	−13.41
NGBDI	0.16	5.81	−6.19	−4.06	0.16	−5.49	−11.34	−9.32	0.16	6.60	12.79	2.28	0.13	4.23	10.28	−2.23
EXG	0.09	1.46	−37.23	−27.83	0.07	−1.44	−38.13	−28.87	0.09	59.31	61.63	14.97	0.04	38.56	40.58	−13.02

### Selection of RGB texture

Table 3 showed the standard deviation and difference coefficient of the RGB texture for different vegetation of study area A. The features to distinguish between *Pinus sylvestris* and maize were determined according to the feature sorting and were the contrast of red band as well as blue band and the variance of blue band. Similarly, the contrast and variance of red band as well as blue band were determined to distinguish between *Pinus sylvestris* and sunflower as well as grassland. The features to distinguish between grassland and maize, *Pinus sylvestris*, sunflower were the variance and contrast of green band as well as red band. The mean of red band, green band and blue band were with larger difference coefficient and less standard deviation to distinguish between sunflower and maize. Finally, the contrast and variance of red band as well as the contrast of blue band were determined to be the optimal features for RGB texture analysis based on the frequency and standard deviation of these features. This paper performed the same actions to the study area B and the result showed that optimal RGB textures were the variance and contrast of green band as well as the contrast of blue band. Thus, it could be seen that the variance and contrast played an important role to distinguish between vegetation for RGB texture.

**Table 3:** Standard deviation and difference coefficient of R, G, and B texture between different vegetation %.

Indexes	Maize				*Pinus sylvestris*				Sunflower				Grass			
	*S*	$D_{W}$ *Pinus sylvestris*	$D_{W}$ Sunflower	$D_{W}$ Grass	*S*	$D_{W}$ Maize	$D_{W}$ Sunflower	$D_{W}$ Grass	*S*	$D_{W}$ Maize	$D_{W}$ *Pinus sylvestris*	$D_{W}$ Grass	*S*	$D_{W}$ Maize	$D_{W}$ *Pinus sylvestris*	$D_{W}$ Sunflower
R Mean	0.16	−9.32	−41.56	−39.53	0.17	10.28	−35.56	−33.32	0.27	71.13	55.17	3.48	0.26	65.38	49.96	−3.36
R Variance	0.29	139.79	−31.28	599.17	0.12	−58.30	−71.34	191.58	0.42	45.51	248.92	917.37	0.04	−85.70	−65.70	−90.17
R Homogeneity	0.20	−33.92	16.43	−53.49	0.30	51.33	76.19	−29.61	0.17	−14.11	−43.24	−60.05	0.42	114.99	42.07	150.31
R Contrast	0.28	158.05	−29.50	570.92	0.11	−61.25	−72.68	160.00	0.39	41.85	266.04	851.72	0.04	−85.10	−61.54	−89.49
R Dissimilarity	0.48	60.62	−16.90	158.50	0.30	−37.74	−48.26	60.94	0.57	20.33	93.27	211.05	0.18	−61.32	−37.87	−67.85
R Entropy	0.98	2.70	−0.31	10.91	0.95	−2.63	−2.92	8.00	0.98	0.31	3.01	11.25	0.88	−9.84	−7.41	−10.12
R Second Moment	0.12	−7.46	0.97	−25.07	0.13	8.06	9.10	−19.03	0.12	−0.96	−8.34	−25.78	0.16	33.45	23.50	34.74
R Correlation	0.16	−16.21	−9.35	8.92	0.55	19.34	8.18	29.99	0.51	10.32	−7.57	20.16	0.42	−8.19	−23.07	−16.78
G Mean	0.18	−3.08	−38.95	−33.33	0.18	3.18	−37.00	−31.21	0.29	63.79	58.73	9.20	0.27	50.00	45.37	−8.42
G Variance	0.36	142.63	−23.52	868.30	0.15	−58.79	−68.48	299.08	0.47	30.76	217.27	1166.15	0.04	−89.67	−74.94	−92.10
G Homogeneity	0.17	−37.49	17.71	−60.69	0.28	59.97	88.30	−37.12	0.15	−15.04	−46.89	−66.61	0.44	154.40	59.03	199.45
G Contrast	0.35	165.30	−20.50	796.47	0.13	−62.31	−70.03	237.90	0.44	25.78	233.71	1027.60	0.04	−88.85	−70.41	−91.13
G Dissimilarity	0.53	63.61	−12.38	203.52	0.32	−38.88	−46.45	85.51	0.60	14.13	86.72	246.39	0.17	−67.05	−46.09	−71.13
G Entropy	0.99	2.33	0.05	13.98	0.96	−2.28	−2.23	11.39	0.99	−0.05	2.28	13.92	0.87	−12.27	−10.22	−12.22
G Second Moment	0.12	−6.78	−0.13	−30.24	0.12	7.27	7.13	−25.17	0.12	0.13	−6.66	−30.15	0.17	43.35	33.63	43.17
G Correlation	0.46	−17.08	−8.86	8.69	0.55	20.60	9.91	31.07	0.50	9.72	−9.02	19.25	0.42	−7.99	−23.71	−16.14
B Mean	0.11	−5.82	−40.75	−37.38	0.11	6.18	−37.09	−33.51	0.18	68.77	58.95	5.68	0.17	59.70	50.41	−5.38
B Variance	0.31	230.53	−19.10	514.82	0.09	−69.75	−75.52	86.01	0.38	23.61	308.56	659.95	0.05	−83.74	−46.24	−86.84
B Homogeneity	0.23	−39.09	25.70	−50.45	0.38	64.17	106.36	−18.66	0.18	−20.44	−51.54	−60.58	0.47	101.82	22.94	153.69
B Contrast	0.31	258.40	−14.14	517.06	0.09	−72.10	−76.04	72.17	0.36	16.47	317.43	618.68	0.05	−83.79	−41.92	−86.09
B Dissimilarity	0.48	87.64	−11.28	150.63	0.25	−46.71	−52.72	33.57	0.54	12.72	111.50	182.50	0.19	−60.10	−25.13	−64.60
B Entropy	0.96	5.06	−1.40	13.96	0.92	−4.81	−6.15	8.48	0.98	1.42	6.55	15.58	0.85	−12.25	−7.81	−13.48
B Second Moment	0.13	−12.98	4.54	−28.75	0.14	14.91	20.13	−18.12	0.12	−4.34	−16.76	−31.84	0.18	40.34	22.13	46.72
B Correlation	0.45	−16.32	−4.53	3.08	0.53	19.50	14.09	23.17	0.47	4.75	−12.35	7.97	0.43	−2.98	−18.81	−7.38

#### Selection of HSV texture.

Table 4 showed the standard deviation and difference coefficient of the HSV texture for different vegetation of study area A. The second moment of brightness could be used to distinguish between maize and *Pinus sylvestris*. The second moment and variance of saturation as well as the second moment and mean of brightness could be used to distinguish between maize and sunflower. The features to distinguish between maize and grassland were the variance of hue and saturation as well as the contrast of hue and brightness. The second moment and contrast of saturation as well as the mean and second moment of brightness were with larger difference coefficient and less standard deviation to distinguish between *Pinus sylvestris* and sunflower. The features to distinguish between the *Pinus sylvestris* and grassland were the contrast of hue and saturation as well as the variance of hue, saturation and brightness. And the contrast and variance of hue and saturation could be used to differentiate between sunflower and grassland. Finally, the contrast of hue, the variance of saturation and the second moment of brightness were determined to be the optimal features for HSV texture analysis based on the frequency and standard deviation of these features. This paper performed the same actions to the study area B and the result showed that optimal HSV textures were the variance of hue as well as the variance and contrast of brightness. Thus, it could be seen that the variance and contrast also played an important role to distinguish between vegetation for HSV texture.

**Table 4:** Standard deviation and difference coefficient of H, S, and V texture between different vegetation %.

Indexes	Maize				*Pinus sylvestris*				Sunflower				Grass			
	*S*	$D_{W}$ *Pinus sylvestris*	$D_{W}$ Sunflower	$D_{W}$ Grass	*S*	$D_{W}$ Maize	$D_{W}$ Sunflower	$D_{W}$ Grass	*S*	$D_{W}$ Maize	$D_{W}$ *Pinus sylvestris*	$D_{W}$ Grass	*S*	$D_{W}$ Maize	$D_{W}$ *Pinus sylvestris*	$D_{W}$ Sunflower
H Mean	0.04	−4.75	−3.31	−7.69	0.06	4.99	1.51	−3.09	0.03	3.43	−1.49	−4.53	0.07	8.34	3.19	4.75
H Variance	0.04	−18.34	−3.28	369.96	0.06	22.46	18.45	475.50	0.03	3.39	−15.57	385.87	0	−78.72	−82.62	−79.42
H Homogeneity	0.13	−8.81	2.95	−28.80	0.15	9.66	12.89	−21.92	0.14	−2.86	−11.42	−30.84	0.11	40.44	28.07	44.58
H Contrast	0.05	−1.95	23.29	553.53	0.06	1.99	25.75	566.52	0.03	−18.89	−20.47	430.05	0	−84.70	−85.00	−81.13
H Dissimilarity	0.09	10.39	0.63	149.62	0.09	−9.41	−8.85	126.13	0.07	−0.62	9.70	148.07	0.03	−59.94	−55.78	−59.69
H Entropy	0.13	3.52	−8.84	58.96	0.16	−3.40	−11.94	53.56	0.14	9.70	13.55	74.37	0.14	−37.09	−34.88	−42.65
H Second Moment	0.09	−18.71	7.49	−54.92	0.14	23.02	32.23	−44.53	0.13	−6.96	−24.38	−58.06	0.22	121.80	80.29	138.41
H Correlation	0.15	−29.87	−37.51	−27.01	0.18	42.59	−10.90	4.07	0.16	60.03	12.23	16.80	0.18	37.01	−3.91	−14.39
S Mean	0.12	−1.93	−37.73	−32.13	0.13	1.97	−36.51	−30.79	0.15	60.60	57.50	9.01	0.13	47.33	44.49	−8.26
S Variance	0.05	5.73	−76.32	268.60	0.06	−5.42	−77.61	248.62	0.36	322.35	346.56	1456.80	0.02	−72.87	−71.32	−93.58
S Homogeneity	0.12	−33.18	14.74	−59.65	0.12	49.65	71.70	−39.61	0.14	−12.84	−41.76	−64.83	0.08	147.81	65.59	184.32
S Contrast	0.08	56.77	−68.13	355.77	0.05	−36.21	−79.67	190.73	0.37	213.75	391.86	1329.97	0.02	−78.06	−65.60	−93.01
S Dissimilarity	0.12	30.35	−30.88	142.82	0.10	−23.28	−46.97	86.28	0.27	44.68	88.59	251.31	0.04	−58.82	−46.32	−71.54
S Entropy	0.08	5.49	−5.09	49.90	0.09	−5.20	−10.03	42.11	0.08	5.37	11.15	57.95	0.08	−33.29	−29.63	−36.69
S Second Moment	0.02	−27.53	36.76	−77.96	0.03	37.98	88.71	−69.59	0.02	−26.88	−47.01	−83.88	0.06	353.68	228.80	520.48
S Correlation	0.17	−37.67	−34.38	−22.49	0.18	60.44	5.28	24.36	0.13	52.40	−5.01	18.12	0.15	29.01	−19.59	−15.34
V Mean	0.05	−6.32	−45.02	−40.82	0.05	6.75	−41.31	−36.83	0.04	81.87	70.37	7.63	0.06	68.98	58.30	−7.09
V Variance	0.17	86.68	−31.50	507.31	0.09	−46.43	−63.31	225.32	0.18	45.99	172.54	786.61	0.03	−83.53	−69.26	−88.72
V Homogeneity	0.12	−46.70	22.90	−61.81	0.11	87.63	130.60	−28.35	0.10	−18.64	−56.64	−68.93	0.07	161.87	39.57	221.85
V Contrast	0.26	177.73	−18.13	554.03	0.08	−63.99	−70.52	135.49	0.21	22.15	239.25	698.90	0.02	−84.71	−57.54	−87.48
V Dissimilarity	0.20	68.41	−11.13	163.33	0.10	−40.62	−47.23	56.36	0.17	12.52	89.50	196.30	0.04	−62.02	−36.04	−66.25
V Entropy	0.07	13.06	−4.47	52.74	0.08	−11.55	−15.50	35.10	0.06	4.68	18.35	59.89	0.07	−34.53	−25.98	−37.46
V Second Moment	0.01	−49.38	43.82	−80.78	0.02	97.57	184.14	−62.03	0.01	−30.47	−64.81	−86.64	0.04	420.27	163.33	648.22
V Correlation	0.15	−41.78	−25.58	−3.85	0.16	71.77	27.82	65.15	0.19	34.38	−21.77	29.20	0.15	4.01	−39.45	−22.60

### Classification results with different feature combinations

**Fig 5:**
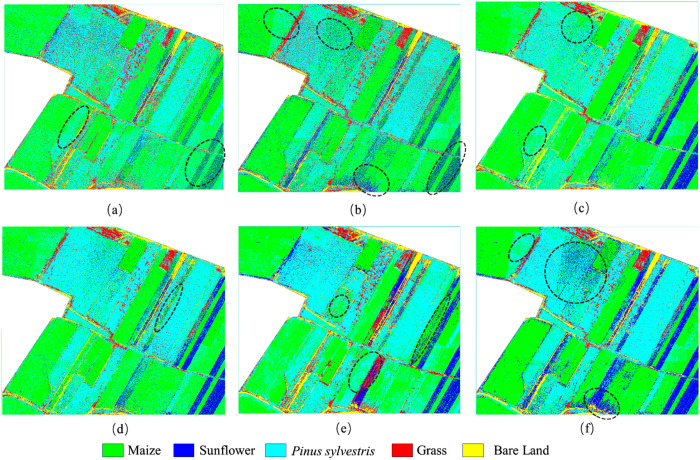
Classification results based on the feature combinations of study area A. (a) RGB& selected VIs; (b) RGB& selected RGB texture; (c) RGB& selected HSV texture; (d) RGB& selected VIs &RGB texture & HSV texture; (e) RGB& selected features by RF1; (f) RGB& selected features by RF2.

This paper performed SVM classification on the feature combinations mentioned above of study area A, and obtained the classification results as shown in Fig 5. It could be observed that all the combinations obtained good identification results for the *Pinus sylvestris* with larger crowns since the obvious differences with other vegetation as indicated by pink circle in Fig 5a. However, the identification varied greatly for the *Pinus sylvestris* with smaller crowns. Among them, there were cases where *Pinus sylvestris* were misclassified as maize for the combinations of RGB with selected VIs as indicated by Fig 5a. Besides, there were more omission cases for sunflowers which could only recognize the flower heads while miss the leaves as marked by Fig 5a. When using the combinations of RGB with selected RGB texture, some maizes were misclassified as *Pinus sylvestris* as indicated in the top left of Fig 5b. This might be due to slight blurriness of this area thereby causing differences of RGB texture compared to that of surrounding area. However, it was not affected for the combinations of RGB with selected VIs. Besides, *Pinus sylvestris* was still misclassified as maize in several areas such as the marked area in the top right of Fig 5b and the same went for the combinations of RGB with selected HSV texture as indicated by Fig 5c. However, sunflower recognition of the third combinations were obviously better than the previous two combinations. For the combinations of RGB with selected VIs, RGB texture and HSV texture, the result could significantly improve for the aforementioned issues, but they were difficult to identify the small sunflowers that had not yet blossomed just like the effects of previous combinations as marked in Fig 5d.

**Fig 6:**
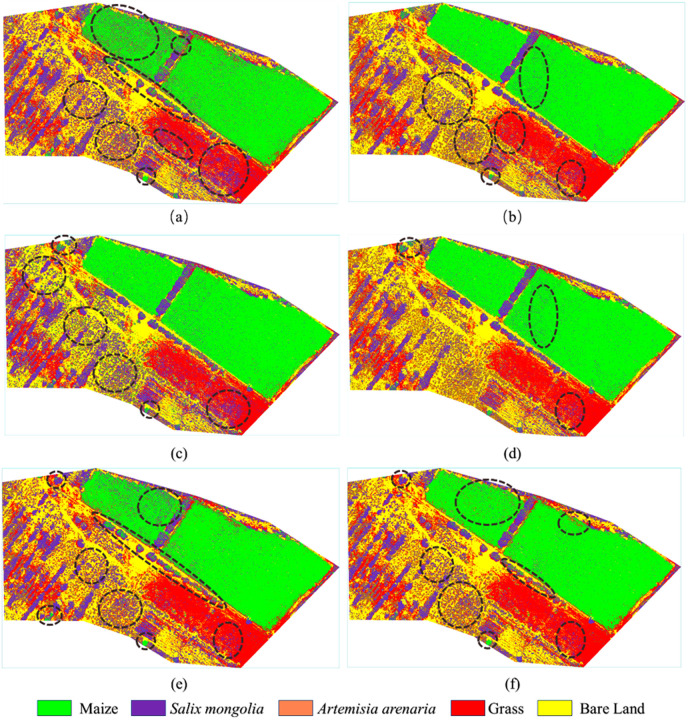
Classification results based on the feature combinations of study area B. (a) RGB& selected VIs; (b) RGB& selected RGB texture; (c) RGB& selected HSV texture; (d) RGB& selected VIs &RGB texture & HSV texture; (e) RGB& selected features by RF1; (f) RGB& selected features by RF2.

The classification results of study area B were as shown in Fig 6. It could be observed that there were misclassification errors between these vegetation for the combinations of RGB image with selected VIs as marked in Fig 6a. Among them, many pixels belonging to the maize and grass were classified into *Salix mongolia*. This situation could improve when combining with the textures. However, there were part misclassification cases between *Artemisia arenaria* and *Salix mongolia* for the combinations of RGB with its texture. The same also went for the combinations of RGB with HSV texture, and there were also the cases that the *Artemisia arenaria* and grass were classified into maize and *Salix mongolia*, respectively. For the combinations of RGB with selected VIs, RGB texture and HSV texture, the result could significantly improve compared with other combinations.

The confusion matrix was used to evaluate the accuracy of vegetation extraction, and validation samples were randomly and uniformly selected on the image through visual interpretation. There was a total of 104021 validation samples for the study area A, including 36518 maize samples, 29633 *Pinus sylvestris* samples, 14289 sunflower samples, 15859 grassland samples, and 7722bare land samples. There was a total of 70315 validation samples, including 20010 maize samples, 24220 *Salix mongolia* samples, 10952 *Artemisia arenaria* samples, 9972 grassland samples, and 5161 bare land samples.

The accuracy evaluation results were as shown in Table 5 and 6. It could be observed that the combinations of RGB& optimal VIs were with the lowest accuracy for both the overall accuracy (OA) and Kappa values, then followed by RGB& optimal textures, and the combinations of RGB& all selected features obtained the highest accuracy. This indicated that the inclusion of VIs was beneficial for the vegetation identification in the heterogenous areas, but they were less important than the texture features.

**Table 5:** Accuracy evaluation of different feature combinations of study area A.

Feature Combinations	Producer accuracy (%)				User Accuracy (%)				Overall accuracy(%)	*Kappa*
Maize	Sunflower	*Pinus* *sylvestris*	Grass	Maize	Sunflower	*Pinus* *sylvestris*	Grass		
RGB& optimal VIs	70.27	31.98	75.96	27.35	60.67	52.85	52.85	66.24	60.93	0.4561
RGB& optimal RGB texture	80.72	52.68	73.96	76.39	70.44	85.34	67.29	85.38	74.32	0.6493
RGB& optimal HSV texture	90.34	86.11	78.12	88.93	79.98	96.44	81.11	94.7	85.46	0.8030
RGB& all above selected feature	87.06	89.68	88.97	88.08	86.63	95.66	80.26	95.92	87.76	0.8351
RGB& selected feature by RF1	73.746	81.59	77.77	41.81	74.45	85.08	65.52	71.43	73.75	0.6432
RGB& selected feature by RF2	89.1	88.03	83.27	85.65	84.19	90.48	80.93	95.37	86.24	0.8145

**Table 6:** Accuracy evaluation of different feature combinations of study area B.

Feature Combinations	Producer accuracy (%)				User Accuracy (%)				Overall accuracy(%)	*Kappa*
	Maize	*Salix mongolia*	*Artemisia arenaria*	Grass	Maize	*Salix mongolia*	*Artemisia arenaria*	Grass		
RGB& optimal VIs	86.67	71.4	42.46	70.51	80	68.59	50.52	83.8	73.08	0.6388
RGB& optimal RGB texture	97.72	82.99	76.28	83.95	95.29	84.81	74.33	93.99	87.33	0.8314
RGB& optimal HSV texture	97.37	83.48	51.99	78.67	89.99	77.32	69.23	93.48	82.90	0.7700
RGB& all above selected feature	97.77	84.12	81.5	85.15	96.38	87.01	75.91	94.12	88.74	0.8505
RGB& selected feature by RF1	91.91	77.11	55.33	80.69	87.71	77.07	60.3	86.84	80.00	0.7332
RGB& selected feature by RF2	94.76	78.77	57.87	83.89	92.28	80.34	59.83	87.05	82.25	0.7638

As to the study area A, the highest OA and Kappa values obtained based on the best combinations were 87.76% and 0.8351, respectively. Among them, all the vegetation obtained high producer accuracy and user accuracy exceeding 80%, in which *Pinus sylvestris* had the lowest user accuracy mainly due to the low distinguishability between maize and *Pinus sylvestris*, resulting in most *Pinus sylvestris* being misclassified as maize. Although the OA difference between the third and fourth combinations was only 2.3%, they had large accuracy difference for some vegetation. For example, the gap of producer accuracy was 10.87% for *Pinus sylvestris* between these two combinations.

As to the study area B, the highest OA and Kappa values obtained based on the best combinations were 88.74% and 0.8505, respectively. Among them, maize was with high producer accuracy and user accuracy exceeding 90%, and *Artemisia arenaria* was with the lowest producer accuracy and user accuracy might due to its spectral similarity and partly spatial overlap with *Salix mongolia*.

### Comparison with other feature selected method

To prove the effectiveness of the difference coefficient-based method, we also adopted widely used RF method for the experiment. We constructed two feature sets through the following ways: (1) selecting features from VIs, RGB texture and HSV texture by RF, respectively, and the number of each kind was equal to that of difference coefficient-based method; (2) integrating VIs, RGB texture and HSV texture, and then selecting 8 features by RF (The best combinations had 8 selected features with difference coefficient-based method). These two feature sets were indicated as “selected feature by RF1”and “selected feature by RF2”. The number of trees and predictor variables was set to 500 and 5 by trial and error. For the study area A, NGBDI, EXG and the mean of red, green, blue, hue, saturation and brightness band were the selected features by RF1. These features including RGRI, mean of blue, hue, saturation and brightness band, correlation of hue and brightness band as well as contrast of brightness band were selected by RF2. For the study area B, the features selected by RF1 were just the same as that of the study area A. These features including NGBDI, mean of red, blue, hue and saturation band as well as the variance, contrast and entropy of hue band were selected by RF2. The selected features were combined with RGB image to perform classification and the results were as shown in Fig 5 and 6, and the accuracy evaluations were as shown in Table 5 and 6. It could be observed that the recognition from RF2 combination was better than that from RF1 combination.

As to the study area A, we could observe that there were large numbers of misclassifications between *Pinus sylvestris* and maize, and omission cases for bloomed sunflowers as indicated in Fig 5e for the classification result from RF1 combination, and thereby leading to lower OA and Kappa with 73.75% and 0.6432. As for the combinations from RF2, it could effectively identify the not yet bloomed sunflower, but it misclassified lots of *Pinus sylvestris* and maize into the sunflower as shown in Fig 5f. Thus, its OA and Kappa was lower than that of the best combination from difference coefficient-based method. As to the study area B, many pixels belonging to maize and grass were classified into *Salix mongolia* as indicated in Fig 6e for the classification result from RF1 combination and thereby leading to lower OA and Kappa with 80.00% and 0.7332. As for the combinations from RF2, there were lots of misclassifications between *Salix mongolia* and *Artemisia arenaria* as shown in Fig 6f, thereby leading to lower accuracy than that of the best combination from difference coefficient-based method.

## Discussion

This paper chosen the more affordable UAV RGB image to map these cover types through constructing feature combination and classifying. VIs, RGB texture and HSV texture were extracted and selected by analyzing the standard deviation and difference coefficient. The result showed that the identification accuracy varied greatly for different combinations. Among them, the accuracy of RGB&VIs was much lower than that of RGB&single type of texture indicating that the texture was more important than the used VIs which was consistent with previous findings on desert vegetation [16]. Besides, this paper made comparisons with other feature combinations selected by widely used RF, and the result showed that our constructed optimal combination was superior to those, which further proved that the proposed method was simple and effective. Notably, the introduction of a band selection method based on standard deviation and difference coefficients provides a streamlined yet robust approach for feature selection, enhancing classification performance in complex environments such as mining areas.

**Fig 7:**
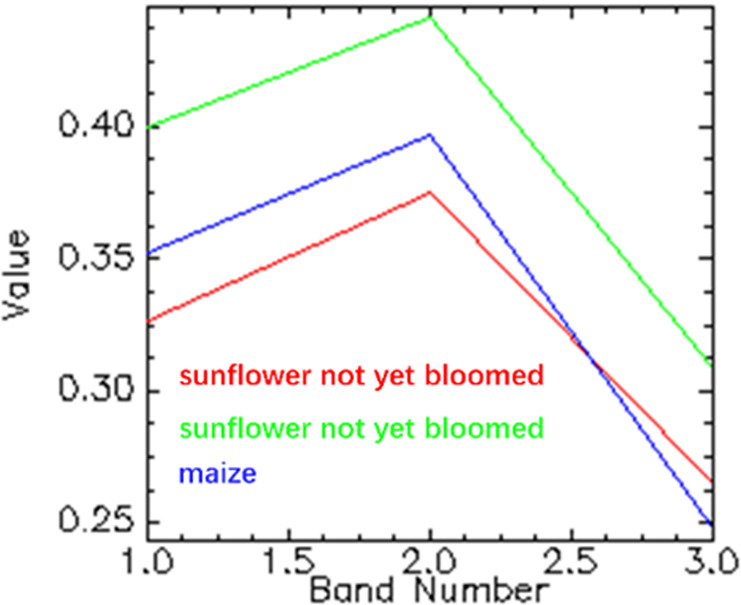
The values of bloomed sunflower, not yet bloomed flower and maize for each band of RGB image.

And there were a lot of factors to impact the vegetation extraction such as growth condition, image quality and spatial heterogeneity. For example, a small part of sunflowers in the study area A had not yet bloomed and showed differences in colors and textures with those bloomed sunflowers which would exacerbate the phenomena of “same object different spectrum” as shown in Fig 7. We could observe that the spectrum of sunflower not yet bloomed (marked by red line) was closer to that of maize (marked by blue line) thereby resulting these sunflowers misclassifying into maize. Besides, longer fight campaigns would increase the probability of encountering weather changes [35] including illumination and wind, therefore influencing the image quality such as decreasing the contrast of ground objects. This might one of the reasons leading to misclassification between *Pinus sylvestris* and maize. Additionally, the changes in the density and scattered distribution would increase the spatial heterogeneity making classification more challenging

It is pointed out that UAV can offer high-frequency observations. Thus, the future research can adopt multi-temporal UAV imagery to leverage the varying phenological characteristics to increase the differentiation between vegetation, thereby further improving the accuracy.

## Conclusion

The vegetation distribution map in mining areas was an important basis for conducting pre-mining ecological assessments and post-mining ecological monitoring. This paper adopted UAV RGB image to identify the vegetation with high heterogeneity, and different feature combinations were constructed based on standard deviation and difference coefficients and then performed with SVM. The results showed that the accuracy for the combinations of RGB& all selected feature were best, next was the combinations of RGB& single type of texture and then was the combinations of RGB &VIs. This indicated that texture features were more important than VIs for vegetation discrimination. The OA and Kappa of best combination were 87.76% and 0.8351 for study area A, whereas 88.74% and 0.8505 for study area B. And the producer accuracy and user accuracy for most vegetation exceeded 80% which indicated that the adopted method was effective for vegetation identification. Besides, our constructed optimal combination was superior to that constructed by RF, which further proved that the proposed method was simple and effective.
